# Active Tuberculosis Is Associated with Depletion of HIV-Specific CD4 and CD8 T Cells in People with HIV

**DOI:** 10.1089/aid.2023.0088

**Published:** 2024-07-11

**Authors:** Jeremiah Khayumbi, Loren E. Sasser, Taryn A. McLaughlin, Benson Muchiri, Joshua Ongalo, Joan Tonui, Samuel Gurrion Ouma, Angie Campbell, Felix Hayara Odhiambo, Chelimo Kiprotich, Neel R. Gandhi, Cheryl L. Day

**Affiliations:** ^1^ Center for Global Health Research, Kenya Medical Research Institute, Kisumu, Kenya.; ^2^ Department of Biomedical Sciences, School of Public Health and Community Development, Maseno University, Kisumu, Kenya.; ^3^ Department of Microbiology and Immunology, Emory Vaccine Center, Emory University School of Medicine, Atlanta, Georgia, USA.; ^4^ Department of Epidemiology, Rollins School of Public Health, Emory University, Atlanta, Georgia, USA.; ^5^ Division of Infectious Diseases, Department of Medicine, Emory University School of Medicine, Atlanta, Georgia, USA.; ^6^ Department of Microbiology and Immunology, Emory University School of Medicine, Atlanta, Georgia, USA.

**Keywords:** HIV, *Mycobacterium tuberculosis*, tuberculosis, CD4 T cell, CD8 T cell, immune activation

## Abstract

Infection with *Mycobacterium tuberculosis* (Mtb) in people with HIV (PWH) is associated with depletion of Mtb-specific CD4 T cell responses, increased risk of progression to active tuberculosis (TB) disease, and increased immune activation. Although higher HIV viral loads have been reported in Mtb/HIV co-infection, the extent to which Mtb infection and TB disease impact the frequency and phenotype of HIV-specific T cell responses has not been well described. We enrolled a cohort of PWH in Kenya across a spectrum of Mtb infection states, including those with no evidence of Mtb infection, latent Mtb infection (LTBI), and active pulmonary TB disease, and evaluated the frequency, immune activation, and cytotoxicity phenotype of HIV-specific CD4 and CD8 T cell responses in peripheral blood by flow cytometry. We found evidence of depletion of HIV-specific CD4 and CD8 T cells in people with TB, but not with LTBI. Expression of the immune activation markers human leukocyte antigen-DR isotype (HLA-DR) and Ki67 and of the cytotoxic molecules granzyme B and perforin were increased in total CD4 and CD8 T cell populations in individuals with TB, although expression of these markers by HIV-specific CD4 and CD8 T cells did not differ by Mtb infection status. These data suggest that TB is associated with overall increased T cell activation and cytotoxicity and with depletion of HIV-specific CD4 and CD8 T cells, which may contribute to further impairment of T cell–mediated immune control of HIV replication in the setting of TB.

## Introduction

Approximately 38.4 million people worldwide are currently living with HIV,^1^ one quarter of whom are estimated to also be infected with *Mycobacterium tuberculosis* (Mtb),^2^ the causative agent of tuberculosis (TB). In 2021, an estimated 10.6 million people were reported ill with TB, with 1.4 million deaths reported from individuals without HIV and an additional 187,000 deaths in people with HIV (PWH).^3^ Infection with HIV is the greatest risk factor for development of active TB, and TB is the leading cause of death in PWH.

Increasing evidence indicates Mtb and HIV coinfection impairs the host's individual disease-containment process and accelerates the progression of both infections.^4,5^ Some studies have reported elevation of HIV plasma viral load^6,7^ as well as increased viral load and viral quasispecies at sites of Mtb infection.^8–10^ HIV in turn impairs macrophage function, thus impairing the ability of macrophages to kill intracellular Mtb.^11–13^ In PWH, plasma levels of immune activation markers are increased in people with latent TB infection (LTBI) and with active TB.^9,14,15^ Together, chronic systemic immune activation, depletion of CD4 T cells, viral load, age, and other underlying mechanisms may contribute to increased risk of progression to TB in PWH. TB is also characterized by high levels of inflammation and immune activation, driven in part by interferon (IFN) signaling,^16^ and there is evidence of continuous inflammation in the setting of asymptomatic LTBI.^17^

Coinfection with Mtb and HIV may therefore further exacerbate systemic immune activation and inflammation,^14,15^ although the extent to which elevated levels of immune activation impact Ag-specific T cell responses in coinfected individuals has not been well described. Although HIV infection has been associated with depletion and functional impairment of Mtb-specific CD4 T cell responses, less is known about the effects of LTBI and TB on the phenotypic and functional profiles of HIV-specific T cell responses, which are essential for control of HIV replication.

Elevated immune activation of T cell responses in PWH has long been recognized to be associated with CD4 T cell depletion, increased viral load, progression to AIDS, and higher risk of mortality.^14,18–21^ TB is associated with increased levels of T cell activation, as measured by human leukocyte antigen-DR isotype (HLA-DR), Ki67, and CD38 expression, compared with LTBI.^22–24^ One previous study of adults in South Africa with chronic HIV infection reported lower frequencies of HIV-specific CD4 and CD8 T cells expressing Th1 cytokines in those with TB, compared with LTBI and those without evidence of Mtb infection,^25^ suggesting HIV-specific T cell functional capacity may be further impaired in the setting of TB. However, this study did not evaluate immune activation or phenotype of HIV-specific T cells and the mechanisms whereby Mtb infection may contribute to impairment of HIV-specific T cell responses remain undefined.

We sought to evaluate the effect of Mtb infection on the frequency, activation, and cytotoxicity phenotype of HIV-specific CD4 and CD8 T cells in PWH across well-defined Mtb infection states. We hypothesized that TB would be associated with increased immune activation and cytotoxicity of HIV-specific CD4 and CD8 T cell responses. To test this hypothesis, we enrolled a cohort of PWH in Kenya across a spectrum of Mtb infection states to evaluate the frequency, absolute count, and phenotype of total and HIV-specific CD4 and CD8 T cells. We found evidence of depletion of HIV-specific CD4 and CD8 T cells in people with TB but not LTBI.

Although markers of immune activation and cytotoxicity were elevated by total CD4 and CD8 T cells in individuals with TB, compared with LTBI and those without Mtb infection, expression of these markers by HIV-specific CD4 and CD8 T cells did not differ significantly in individuals with LTBI and TB, compared with those without evidence of Mtb infection. These data suggest that high bacterial burden in TB may contribute to increased immune activation and depletion of HIV-specific T cell responses, factors that may be attributable to poor immune control of HIV in coinfected individuals.

## Materials and Methods

### Study participants and sample collection

Participants aged ≥18 years were recruited in Kisumu, Kenya as described previously.^26^ Asymptomatic individuals with no previous history of TB disease or treatment were evaluated by an IFN-γ release assay (IGRA) (QuantiFERON-TB Gold In-Tube; Qiagen): those with a negative IGRA result (TB Ag–Nil <0.35 IU IFN-γ/mL) were considered Mtb-uninfected; those with a positive IGRA result (TB Ag–Nil >0.35 IU IFN-γ/mL) were considered to have LTBI. Sputum samples were collected from all participants for testing of Mtb by smear microscopy, Xpert MTB/RIF (Cephid), and liquid culture in BACTEC MGIT mycobacterial growth indicator tubes (BD). All Mtb-uninfected and LTBI participants were negative for acid-fast bacilli by smear microscopy, negative for Mtb complex DNA by Xpert MTB/RIF, and negative for Mtb growth by MGIT liquid culture. Participants with TB symptoms and a positive Xpert MTB/RIF result and a positive MGIT culture for Mtb growth were classified as active TB.^27^

All participants with TB had drug-sensitive TB as determined by GenoType MTBDR*plus*™ (Hain Lifescience). Serologic testing for HIV-1 antibodies was carried out for all individuals using the rapid diagnostic kit for HIV (1 + 2) antibody V2 (*KHB*^®^ Shanghai Kehua Bio-engineering Co., Ltd.); all participants were positive for HIV-1 antibodies. We enrolled a total of 70 participants with HIV, including 23 IGRA^−^, 23 IGRA^+^, and 24 TB, between October 2014 and May 2017. Blood was collected from IGRA^−^ and IGRA^+^ individuals before initiation of antiretroviral therapy (ART). A subset of participants with TB were receiving ART at the time of enrollment in the study (*n* = 7) and are denoted as HIV^+^ TB on ART. All participants not yet on ART were referred to local public health clinics providing HIV care for evaluation and provision of ART according to Kenya Ministry of Health guidelines.

Participants with TB were enrolled within 7 days of initiating the 6-month standard course TB treatment, which was provided according to Kenya Ministry of Health guidelines. Blood was collected from participants in sodium heparin vacutainer tubes (BD Biosciences) for isolation of peripheral blood mononuclear cells (PBMCs) by density centrifugation. PBMCs were cryopreserved and stored in liquid nitrogen until use.

### Ethical consideration

All participants provided written informed consent for the study, which was approved by the KEMRI/CDC Scientific and Ethics Review Unit and the Emory University Institutional Review Board.

### Antigens

HIV-1 Consensus A Gag peptide pool and human cytomegalovirus (CMV) pp65 peptide pool were obtained through the NIH AIDS Reagent Program, Division of AIDS, NIAID, NIH. Staphylococcal enterotoxin B (SEB; 1 μg/mL; Toxin Technology, Inc.) was used as a positive control.

### Antibodies

The following human monoclonal fluorescently conjugated antibodies were used in this study: CD3 AF700 (clone UCHT-1), CD4 BV570 (clone RPA-T4), HLA-DR BV605 (clone L243), CD38 PE-Dazzle 594 (HIT2), Granzyme B PE (clone QA16A02), Ki67 BV711 (clone Ki67), Perforin APC (clone B-D48), CD40L BV421 (clone TRAP1), CD107a PE-Cy7 (H4A3), CD8 PerCP-Cy5.5 (clone SK-1), IFN-γ BV480 (clone B27), and active caspase 3 FITC (clone C92–605). All antibodies were obtained from either BioLegend or BD Biosciences.

### Phenotyping and intracellular cytokine staining assays

Cryopreserved PBMCs were thawed in a 37°C water bath and immediately added to Roswell Park Memorial Institute (RPMI) 1640 medium (Cellgro) supplemented with deoxyribonuclease I (DNase; 10 μg/mL; Sigma-Aldrich). Cells were washed twice in RPMI and suspended in R10 medium (RPMI-1640 supplemented with 10% heat-inactivated fetal calf serum, 100 μg/mL penicillin, 100 μg/mL streptomycin, and 2 mM l-glutamine) and rested for a minimum of 3 h at 37°C and 5% CO_2_. Pure functional grade anti-CD40 antibody (0.5 μg/mL; Miltenyi Biotec) was added to the cells 15 min before addition of CD107a PE-Cy7 antibody and either HIV-1 Gag clade A peptide pool (1 μg/mL) or CMV pp65 peptide pool (1 μg/mL). Cells incubated in R10 media alone served as a negative control (unstimulated) and cells stimulated with SEB (1 μg/mL) served as a positive control. After 1 h, Brefeldin A (10 μg/mL; Fisher Scientific) and monensin (BioLegend) were added and the incubation continued overnight at 37°C and 5% CO_2_.

After stimulation, cells were washed with phosphate-buffered saline (PBS) and stained with the Fixable Viability Dye Zombie Near-IR (BioLegend) for 15 min at room temperature. Samples were then surface stained for 30 min at room temperature with the following antibodies: CD3 AF700, CD4 BV570, CD8 PerCP-Cy5.5, HLA-DR BV605, and CD38 PE/Dazzle 594. Cells were then fixed and permeabilized using BD FACSLysing solution and Perm Wash buffer (BD Biosciences) and stained for intracellular markers on ice for 30 min with the following antibodies: granzyme B PE, IFN-γ BV480, Ki67 BV711, perforin APC, CD40L BV421, and active caspase 3 FITC. Finally, cells were washed in Perm Wash buffer and suspended in 300 μL PBS before acquisition on a BD LSRII flow cytometer with BD FACSDiva software (v8.0).

### Data analysis and statistics

Flow cytometry data were analyzed using FlowJo^®^ version 10.8. Compensation was calculated using single-stained anti-mouse Ig,κ Comp Beads (BD Biosciences). Single cells were gated after plotting forward scatter area versus forward scatter height, whereas lymphocytes were gated based on morphological characteristics by plotting forward scatter area against side scatter area. Viable cells were defined as Zombie Near-IR^lo^ cells. CD4 T cells were defined as CD3^+^CD4^+^CD8^−^ lymphocytes, whereas CD8 T cells were defined as CD3^+^CD4^−^CD8^+^. Ag-specific CD4 T cells were defined as IFN-γ^+^ and/or CD40L^+^ cells; Ag-specific CD8 T cells were defined as IFN-γ^+^ and/or CD107a^+^ following stimulation with Gag peptide pool. The flow cytometry gating strategy is given in Supplementary Figure S1.

Background expression of IFN-γ, CD40L, and CD107a in the negative control condition was subtracted from peptide pool-stimulated conditions when determining Ag-specific CD4 and CD8 T cell frequencies. The absolute number of Ag-specific CD4 and CD8 T cells was calculated by multiplying the absolute CD4 and absolute CD8 T cell counts by the frequency of Ag-specific cells after background subtraction. Mixture models for single-cell assays (MIMOSA)^28^ was used to identify positive responses to Gag peptide pool stimulation for phenotypic analysis of HIV-specific CD4 and CD8 T cell responses. Samples with a probability of response >80% and a false discovery rate (fdr/*q* value) <5% by MIMOSA were considered positive, with phenotypic analysis of HIV-specific CD4 and CD8 T cells restricted to individuals who met the criteria for a positive response.

GraphPad Prism version 6 was used to perform statistical analyses. Differences between three groups were evaluated using a nonparametric Kruskal–Wallis test, followed by pairwise comparisons between two groups using Dunn's multiple comparisons test. Values of *p* < .05 were considered significant.

## Results

### Study participants

Blood samples were collected from PWH who were recruited and enrolled in Kisumu, Kenya. Participants were adults ≥18 years of age and categorized into three groups based on their Mtb infection status: without Mtb infection (IGRA^−^), LTBI (IGRA^+^), and active TB. A subset of participants with TB were receiving ART and are denoted as HIV^+^ TB on ART. Participants with TB who were not on ART had lower CD4 counts than IGRA^−^ and IGRA^+^ participants and higher HIV viral loads than IGRA^+^ participants. The participant groups did not differ otherwise by age and sex (Table 1).

**Table 1.** Participant Characteristics

Participant group	*n*	Age, years*^a^* (IQR)	Sex (% female)	CD4 count, cells/μL*^b^* (IQR)	HIV viral load, copies RNA/mL*^b^* (IQR)	% on ART	% Culture^+^ for Mtb growth	Days to Mtb culture positivity (IQR)*^b^*
HIV^+^ IGRA^–^	23	30 (25–47)	83	502 (427–589)^*^	29,216 (6,794–82,168)	0	0	N/A
HIV^+^ IGRA^+^	23	33 (24–39)	57	577 (439–736)^**^	14,106 (3,066–36,820)^*^	0	0	N/A
HIV^+^ TB	17	36 (31–41)	47	226 (89–366)	105,862 (29,384–376,951)	0	100	6 (5–10)
HIV^+^ TB on ART	7	33 (31–42)	71	282 (91–303)	20 (20–328)	100	100	6 (4–12)

^*^ *p* < .01, compared with HIV^+^ TB.

^**^ *p* < .0001, compared with HIV^+^ TB.

^a^ Values denote median age in years.

^b^ Values denote median.

ART, antiretroviral therapy; IGRA, IFN-γ release assay; IQR, interquartile range; N/A, not applicable; TB, tuberculosis.

### HIV-specific CD4 T cells are depleted in people with TB

**FIG. 1.**
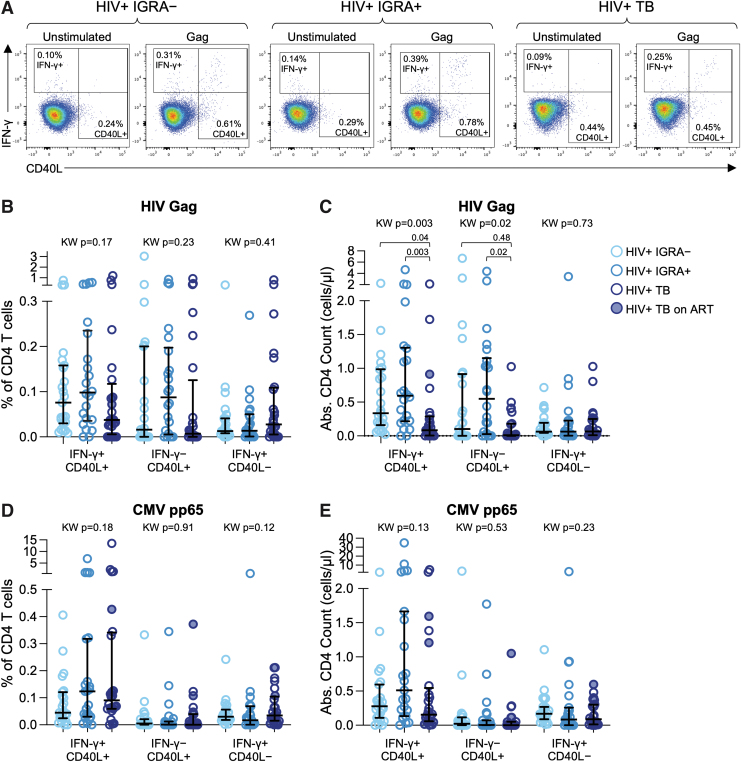
HIV-specific CD4 T cells are depleted in people with active TB. PBMCs were stimulated overnight with HIV-1 Gag Clade A peptide pool and CMV pp65 peptide pool and analyzed by multi-parameter flow cytometry using an intracellular cytokine staining assay. **(A)** Representative flow cytometry plots indicating IFN-γ and CD40L expression by CD4 T cells. Plots are shown gated on live CD4 T cells. **(B)** Frequencies of HIV-specific CD4 T cells expressing the indicated combinations of IFN-γ and CD40L. **(C)** Absolute number of HIV-specific CD4 T cells expressing the indicated combinations of IFN-γ and CD40L. **(D)** Frequencies of CMV-specific CD4 T cells expressing the indicated combinations of IFN-γ and CD40L. **(E)** Absolute number of CMV-specific CD4 T cells expressing the indicated combinations of IFN-γ and CD40L. Data in **(B)** and **(D)** are shown after subtraction of background IFN-γ and CD40L expression in the negative control condition. The median and interquartile range is indicated in each dataset in **(B–E)**. Differences between the three groups in **(B–E)** were first evaluated using a nonparametric Kruskal–Wallis test; comparisons with Kruskal–Wallis *p* < .05 were further evaluated by pairwise comparisons between two groups using Dunn's multiple comparisons test. CMV, cytomegalovirus; IGRA, IFN-γ release assay; PBMCs, peripheral blood mononuclear cells; TB, tuberculosis.

To evaluate the effect of Mtb infection status on the frequency and absolute count of HIV-specific CD4 T cells, we stimulated PBMC with a pool of HIV-1 Gag Clade A peptides followed by intracellular staining for IFN-γ and CD40L and acquisition on a multiparameter flow cytometer (Fig. 1A). The percentage of CD4 T cells expressing IFN-γ and/or CD40L did not differ significantly by Mtb status (Fig. 1B). However, the absolute numbers of HIV-specific IFN-γ^+^CD40L^+^ and IFN-γ^−^CD40L^+^ CD4 T cells were significantly lower in people with TB (Fig. 1C). There were no differences in the absolute counts of HIV-specific CD4 T cells between IGRA^–^ and IGRA^+^ individuals, suggesting Mtb infection alone is insufficient to drive depletion of HIV-specific CD4 T cells.

To further investigate the relevance of antigen specificity in CD4 T cell depletion in people with TB, we evaluated the frequency and absolute counts of CMV-specific CD4 T cells in each participant group. We found no significant differences in the frequencies of CMV-specific CD4 T cells expressing any combination of IFN-γ and CD40L across the three participant groups (Fig. 1D). Moreover, we found no evidence of decreased absolute counts of CMV-specific CD4 T cells in people with TB, compared with IGRA^–^ and IGRA^+^ individuals (Fig. 1E), suggesting that HIV-specific CD4 T cells may be preferentially depleted in people with TB.

### HIV-specific CD8 T cells are depleted in people with TB

**FIG. 2.**
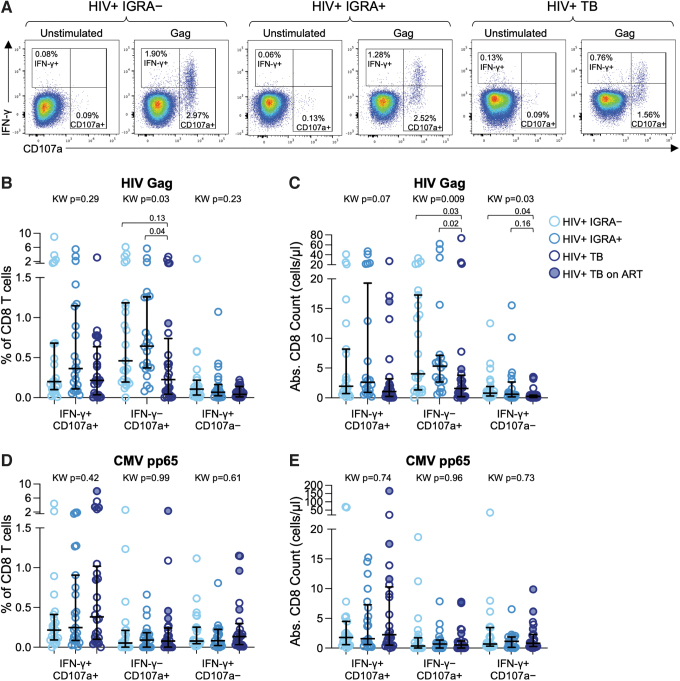
HIV-specific CD8 T cells are depleted in people with active TB. PBMCs were stimulated with HIV-1 Gag Clade A peptide pool and CMV pp65 peptide pool as described in Figure 1. **(A)** Representative flow cytometry plots indicating IFN-γ and CD107a expression by CD8 T cells. Plots shown are gated on live CD8 T cells. **(B)** Frequencies of HIV-specific CD8 T cells expressing the indicated combinations of IFN-γ and CD107a. **(C)** Absolute number of HIV-specific CD8 T cells producing the indicated combinations of IFN-γ and CD107a. **(D)** Frequencies of CMV-specific CD8 T cells expressing the indicated combinations of IFN-γ and CD107a. **(E)** Absolute number of CMV-specific CD8 T cells producing the indicated combinations of IFN-γ and CD107a. Data in **(B)** and **(D)** are shown after subtraction of background IFN-γ and CD107a expression in the negative control condition. The median and interquartile range is indicated in each dataset in **(B–E)**. Differences between the three groups in **(B–E)** were first evaluated using a nonparametric Kruskal–Wallis test; comparisons with Kruskal–Wallis *p* < .05 were further evaluated by pairwise comparisons between two groups using Dunn's multiple comparisons test.

CD4 T cells have long been recognized to provide help to CD8 T cells, which are essential in control of HIV. Given evidence for loss of CD4 T cells in people with TB, we next evaluated the impact of Mtb infection and TB disease on the frequency and absolute count of HIV-specific CD8 T cells. HIV-specific CD8 T cells were identified by intracellular expression of IFN-γ and by upregulation of surface CD107a following stimulation with HIV Gag Clade A peptide pool (Fig. 2A). Robust HIV-specific CD8 T cell responses were detected in all three groups, with a lower frequency of cytotoxic IFN-γ^–^CD107a^+^ CD8 T cells in people with TB, compared with IGRA^+^ individuals (Fig. 1B). The absolute number of HIV-specific IFN-γ^–^CD107a^+^ CD8 T cells was also significantly lower in people with TB, compared with IGRA^–^ and IGRA^+^ individuals (Fig. 1C).

Although IFN-γ^+^CD107a^–^ cells represented the lowest proportion of HIV-specific CD8 T cells, this subset was also depleted in people with TB (Fig. 1C). Similar to HIV-specific CD4 T cells, there were no differences in HIV-specific CD8 T cell frequency and absolute count between IGRA^–^ and IGRA^+^ individuals, suggesting HIV-specific CD8 T cell depletion occurs in people with TB but not with LTBI. In addition, the frequencies and absolute counts of CMV-specific CD8 T cells expressing any combination of CD107a and IFN-γ did not differ by Mtb infection status (Fig. 2D, E), thus providing further evidence that HIV-specific CD8 T cells may be preferentially depleted in people with TB.

### Active TB is associated with increased activation and cytotoxic phenotype of total CD4 and CD8 T cells but not HIV-specific CD4 and CD8 T cells

**FIG. 3.**
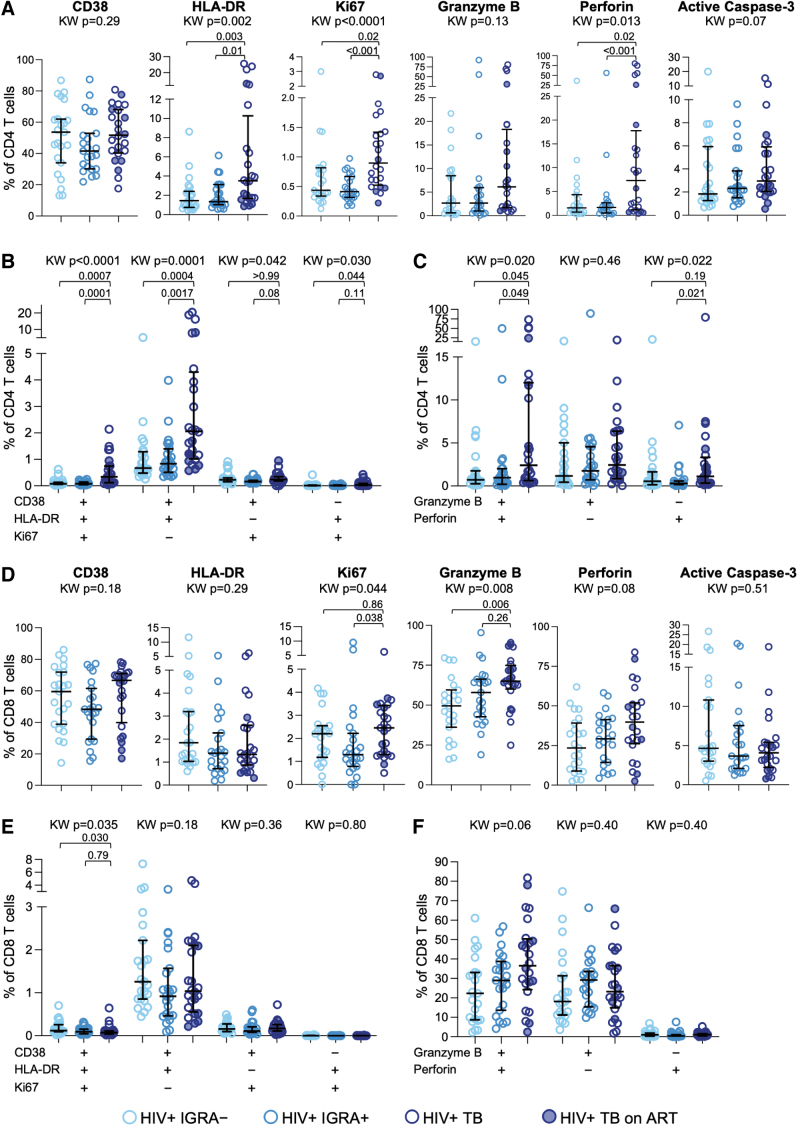
Active TB is associated with increased activation and cytotoxic phenotype of CD4 and CD8 T cells in PWH. CD4 and CD8 T cells in PBMCs were evaluated by flow cytometry for expression of markers associated with immune activation, apoptosis, and cytotoxicity. Expression of each indicated phenotypic marker was evaluated on CD4 and CD8 T cells from the three groups. **(A)** Composite data of the percentage of CD4 T cells expressing each indicated marker. **(B)** Percentage of CD4 T cells expressing the indicated combination of two or more activation markers. **(C)** Percentage of CD4 T cells expressing the indicated combinations of granzyme B and perforin. **(D)** Composite data of the percentage of CD8 T cells expressing each indicated marker. **(E)** Percentage of CD8 T cells expressing the indicated combination of two or more activation markers. **(F)** Percentage of CD8 T cells expressing the indicated combinations of granzyme B and perforin. The median and interquartile range are indicated on each graph. Differences in expression of each marker by CD4 and CD8 T cells in the three groups of PWH were first evaluated using a nonparametric Kruskal–Wallis test; comparisons with Kruskal–Wallis *p* < .05 were further evaluated by pairwise comparisons between two groups using Dunn's multiple comparisons test. PWH, people with HIV.

To determine whether depletion of HIV-specific CD4 and CD8 T cells in people with TB is associated with altered phenotypic profiles, we evaluated expression of activation markers (CD38, HLA-DR, and Ki67), apoptosis (active caspase-3), and cytotoxicity (granzyme B and perforin) on CD4 and CD8 T cells. CD4 T cells in TB participants exhibited significantly higher expression HLA-DR, Ki67, and perforin (Fig. 3A). We next evaluated the coexpression of two or more activation markers and found significantly higher frequencies of CD38^+^HLA-DR^+^Ki67^+^ and CD38^+^HLA-DR^+^Ki67^–^ CD4 T cells in individuals with TB, compared with IGRA^–^ and IGRA^+^ individuals (Fig. 3B). The frequency of CD4 T cells coexpressing granzyme B and perforin was also significantly higher in individuals with TB, compared with IGRA^–^ and IGRA^+^ individuals (Fig. 3C).

CD8 T cells in TB displayed higher expression of Ki67 and granzyme B, compared with IGRA^–^ and IGRA^+^ individuals (Fig. 3D). However, unlike CD4 T cells, we did not find evidence of increased frequencies of CD8 T cells coexpressing two or more activation markers (Fig. 3E) or increased frequencies of CD8 T cells coexpressing granzyme B and perforin (Fig. 3F) in people with TB, compared with IGRA^–^ and IGRA^+^ individuals. These data suggest increased activation and cytotoxicity is more pronounced in CD4 T cells than CD8 T cells in people with TB.

Expression of active caspase 3 did not differ among the three groups for either CD4 or CD8 T cells. No differences in any of the phenotypic markers evaluated were found between IGRA^–^ and IGRA^+^ individuals, suggesting that LTBI alone is not sufficient to modify the activation and cytotoxic phenotype of total CD4 and CD8 T cells in PWH.

**FIG. 4.**
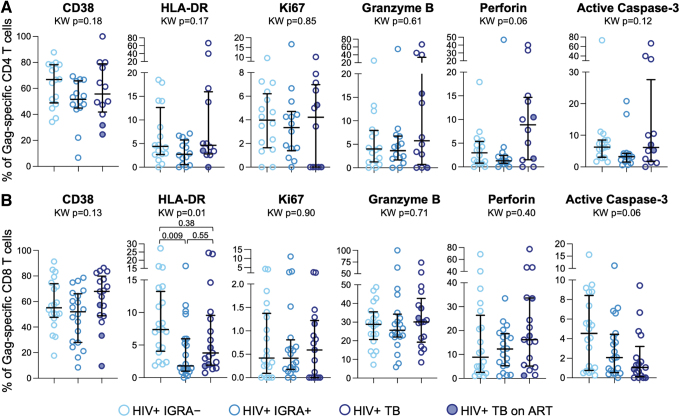
Activation, apoptosis, and cytotoxicity phenotype of HIV-specific CD4 and CD8 T cells are not substantially modified by Mtb infection and TB disease. PBMCs were stimulated with HIV-1 Clade A Gag peptide pool as described in Figure 1. The proportion of IFN-γ^+^ HIV-specific CD4 **(A)** and CD8 T cells **(B)** expressing each indicated marker is shown. The median and interquartile range are indicated on each graph. Differences between the three groups in **(A)** and **(B)** were first evaluated using a nonparametric Kruskal–Wallis test; comparisons with Kruskal–Wallis *p* < .05 were further evaluated by pairwise comparisons between two groups using Dunn's multiple comparisons test.

Finally, we evaluated the phenotype of HIV-specific CD4 and CD8 T cells expressing markers of immune activation, apoptosis, and cytotoxicity. We found no differences in the proportion of HIV-specific CD4 T cells expressing CD38, HLA-DR, Ki67, active caspase-3, granzyme B, or perforin (Fig. 4A). Similarly, no significant differences in expression of the same markers were found on HIV-specific CD8 T cells among the three groups (Fig. 4B). Although expression of HLA-DR was elevated on HIV-specific CD8 T cells from IGRA^–^ compared with IGRA^+^ individuals, there was no evidence of increased HLA-DR expression by HIV-specific CD8 T cells in people with TB (Fig. 4B). Taken together, these data indicate that despite evidence of increased immune activation and cytotoxicity on total CD4 and CD8 T cells in PWH and TB, these phenotypic modifications are not evident on HIV-specific CD4 and CD8 T cell populations and suggest that mechanisms other than immune activation and apoptosis may underpin the depletion of HIV-specific CD4 and CD8 T cells in PWH and TB.

## Discussion

In this study, we enrolled cohorts of PWH with well-characterized Mtb infection and TB disease states to test the hypothesis that Mtb/HIV coinfection is associated with heightened levels of immune activation that may adversely impact the frequency and phenotypic profiles of HIV-specific CD4 and CD8 T cells. We found evidence of depletion of HIV-specific CD4 and CD8 T cells in participants with TB, compared with IGRA^+^ and IGRA^–^ participants. Depletion of HIV-specific CD4 T cells was evident among cells expressing CD40L, whereas HIV-specific CD8 T cell depletion was most evident within cytotoxic cells that expressed CD107a in the absence of IFN-γ production.

Of importance, we did not find evidence of depletion of CMV-specific CD4 and CD8 T cells within the same individuals, thus suggesting HIV-specific T cells may be preferentially targeted for depletion in people with TB. Despite evidence of increased immune activation and cytotoxicity by total CD4 and CD8 T cells in participants with active TB, the activation and cytotoxicity phenotypic profile of HIV-specific CD4 and CD8 T cell responses did not differ by Mtb infection and TB disease status.

Although the percentage of HIV-specific CD4 T cells did not differ significantly by Mtb infection state, we did find evidence of depletion of the absolute number of HIV-specific CD4 T cells in participants with TB, particularly within the subset of cells expressing CD40L. Upregulation of CD40L on CD4 T cells is indicative of the capacity to provide costimulation to antigen-presenting cells expressing CD40^29^ and plays an important role in inducing chemokines and HIV-specific antibody and CD8 T cell responses necessary for control of HIV infection.^30^ Our findings of depletion of CD40L^+^ HIV-specific CD4 T cells in people with TB are consistent with previous findings of depletion of CD40L^+^ CD4 T cells in people with advanced HIV disease and CD4 counts <200 cells/μL.^31^ The depletion of Ag-specific CD40L^+^ CD4 T cells in PWH and TB may thus contribute to further functional impairment of the immune response in coinfected individuals.

In addition to depletion of HIV-specific CD4 T cells, we also found evidence of depletion of cytotoxic HIV-specific CD8 T cells in PWH with TB. Loss of HIV-specific CD8 T cells in PWH has been associated with failure of T cell immunity and HIV disease progression.^32^ Moreover, HIV-specific CD8 T cells with cytotoxic capacity have been associated with enhanced killing of autologous HIV-infected CD4 T cells and slower HIV disease progression.^33–36^ Depletion of HIV-specific CD8 T cells with cytotoxic capacity in people with TB may lead to poorer CD8 T cell–mediated control of HIV replication, which may contribute to increased HIV viral loads that have been described in people with TB.^6,7^

The lack of evidence of depletion of CMV-specific CD4 and CD8 T cells in people with TB suggests that HIV-specific cells may be preferentially targeted for depletion. Future studies evaluating the frequency and absolute count of CD4 and CD8 T cells specific for a broader range of pathogens are necessary to better define the relevance and role of antigen specificity in driving depletion of distinct T cell subsets in PWH who develop TB.

Lower numbers of HIV-specific CD4 and CD8 T cells in PWH with TB in our Kenyan cohort is consistent with a previous study of South African adults with HIV, which reported decreased frequencies of HIV-specific CD4 and CD8 T cells expressing Th1 cytokines (IFN-γ, tumor necrosis factor, or interleukin 2) in participants with TB, compared with participants with either LTBI or no evidence of Mtb infection.^25^ Of importance, our findings and that of the previous study of PWH in South Africa indicate that LTBI alone, in the absence of active TB disease, is insufficient to drive depletion of HIV-specific T cells, as the frequency and absolute number of HIV-specific CD4 and CD8 T cells did not differ between IGRA^–^ and IGRA^+^ participants.

Elevated immune activation in the setting of both HIV and TB disease and its association with disease progression has been well described. Given our findings of depletion of HIV-specific CD4 and CD8 T cells in people with TB, we hypothesized that elevated immune activation in people with TB may contribute to increased susceptibility to cell death of HIV-specific T cells. Sullivan et al. have evaluated markers of immune activation on total CD4 and CD8 T cell populations in adults and reported increased frequencies of CD38^+^HLA-DR^+^ CD4 and CD8 T cells in PWH with active TB, compared with PWH with LTBI and with no evidence of Mtb infection.^14^ Our findings of increased frequencies of HLA-DR^+^CD38^+^Ki67^+^ and HLA-DR^+^CD38^+^Ki67^–^ and CD4 T cells in people with TB are consistent with this previous report.

Moreover, our findings of increased expression of Ki67 by CD8 T cells in TB, compared with IGRA^–^ and IGRA^+^, provide further evidence of increased immune activation of CD4 and CD8 T cells in PWH with TB.^7,14^ Of note, elevated levels of activation on CD4 and CD8 T cells in our study was evident in people with active TB, but not IGRA^+^ individuals. Future longitudinal studies of PWH before development of TB will be important to determine whether increased expression of HLA-DR, CD38, and Ki67 can be leveraged as a biomarker to facilitate identification of individuals in the early phases of Mtb reactivation and TB progression to prioritize TB testing and initiation of treatment.

Despite evidence of increased immune activation on total CD4 and CD8 T cells in people with TB, we did not find evidence of increased expression of immune activation markers on HIV-specific T cells, thus suggesting that bystander immune activation of HIV-specific T cells may not be directly contributing to depletion of these cells in people with TB. We utilized Ag-induced IFN-γ expression to identify HIV-specific T cells for our phenotypic analyses; hence, HIV-specific T cells that lack IFN-γ production capacity in this assay were not evaluated. Although we evaluated expression of activation markers, we did not evaluate expression of exhaustion markers associated with T cell dysfunction, including PD-1, TIGIT, Tim-3, CTLA-4, and LAG-3, all of which have been reported to be upregulated on T cells in people with HIV.^37^

Our analysis of HIV-specific T cells was also limited to PBMCs, and it is possible that some HIV-specific T cells have trafficked to the site of TB disease in the lung and are thus present at lower frequencies in peripheral blood. Evaluation of the number of HIV-specific T cells in people with TB before and after completion of anti-TB treatment, as well as evaluation of HIV-specific T cells in bronchoalveolar lavage, may provide further clarity on depletion versus compartmentalization of these cells to the lung in people with TB.

Further studies are warranted to elucidate mechanisms driving depletion of HIV-specific T cells in the setting of active TB, which may offer opportunities for intervention to prevent the loss of HIV-specific T cell responses in people with Mtb/HIV coinfection. Increased susceptibility to apoptosis may contribute to T cell depletion and high expression of caspase-3 has previously been reported in people with TB.^38^ Although we did not find evidence of increased expression of active caspase 3 by HIV-specific T cells, prior studies have reported increased caspase-8 activity in PWH, which was linked with necroptosis and increased cell death.^39^ Further evaluation of expression of genes in apoptotic and necrotic signaling pathways may uncover additional mechanisms driving depletion of HIV-specific T cells in people with TB. Single cell transcriptional profiling of HIV-specific T cells in individuals across a spectrum of Mtb infection states will also be important to define mechanistic pathways underpinning depletion of these cells.

Moreover, future studies evaluating the frequency and absolute number of HIV-specific T cells in virally suppressed individuals on long-term ART will be important to better understand the contribution of ongoing viral replication and immune activation to depletion of HIV-specific T cells in people who develop TB.

Strengths of this study include the study cohort, which was well characterized for Mtb infection status, including collection of sputum from all participants for testing by smear microscopy, nucleic acid amplification (Xpert MTB/RIF), and liquid culture (MGIT). The high prevalence of subclinical TB in PWH^40^ presents a challenge in clearly distinguishing Mtb infection and TB disease states in PWH. Sputum samples from all IGRA^–^ and IGRA^+^ individuals were negative for Mtb by smear microscopy, Xpert MTB/RIF, and MGIT culture, thus reducing the likelihood of subclinical TB disease among participants in these groups. There are limitations in this study, including cross-sectional sample collection and lack of availability of longitudinal samples before and after completion of anti-TB treatment in those participants with TB to determine whether HIV-specific T cell responses are restored following bacterial clearance.

The absolute CD4 counts were lower in participants with TB, compared with IGRA^–^ and IGRA^+^ individuals, thus making it difficult to disentangle the effect of active TB disease from HIV-associated immunosuppression in people with low CD4 counts. The majority of participants with TB were evaluated before initiation of ART, thus it remains unclear whether HIV-specific T cells are more preserved in virally suppressed people with TB, compared with viremic individuals. Future studies evaluating HIV-specific T cells in people with Mtb coinfection who initiate ART early after acquisition of HIV will be important to determine whether ART-mediated viral suppression mitigates depletion of HIV-specific T cells in people who develop active TB disease.

We evaluated CD4 and CD8 T cell responses to a pool of overlapping peptides corresponding to the HIV-1 Gag protein sequence; however, it is possible that T cell responses to other HIV proteins may exhibit differential frequency, absolute number, and phenotype across different Mtb infection and TB disease states. The number of participants in each group was small, thus the findings will require further validation in larger cohort studies as well as longitudinal studies to determine whether loss of HIV-specific T cells precedes progression to active TB and whether there is evidence of restoration of HIV-specific T cell responses after completion of anti-TB treatment. Although we evaluated T cell expression of a subset of immune activation and cytotoxicity markers, it is possible that other phenotypic markers, such as markers of exhaustion, senescence, and apoptosis, are differentially expressed by HIV-specific CD4 and CD8 T cells that may be contributing to loss of these cells in people with TB.

In summary, we evaluated the frequency and phenotype of HIV-specific CD4 and CD8 T cell responses in Kenyan adults with HIV who were well characterized for Mtb infection and TB disease status. We found evidence of depletion of HIV-specific CD4 T cells expressing CD40L and depletion of HIV-specific CD8 T cells with cytotoxic capacity in PWH with TB, compared with IGRA^–^ and IGRA^+^ PWH with no history of TB diagnosis or treatment. These data indicate that TB is associated with loss of HIV-specific CD4 and CD8 T cells, which may contribute to further impairment of T cell–mediated immune control of HIV replication in the setting of active TB disease.

## Supplementary Material

Supplementary Figure S1

## References

[1] UNAIDS. Global HIV & AIDS statistics—Fact sheet. 2022. Available from: https://www.unaids.org/en/resources/fact-sheet [Last accessed: June 27, 2023].

[2] Houben RM, Dodd PJ. The Global Burden of Latent Tuberculosis Infection: A Re-estimation Using Mathematical Modelling. PLoS Med 2016;13(10):e1002152; doi: 10.1371/journal.pmed.1002152 27780211 PMC5079585

[3] World Health Organization. Global Tuberculosis Report 2022. 2022. Available from: https://www.who.int/teams/global-tuberculosis-programme/tb-reports/global-tuberculosis-report-2022 [Last accessed: June 27, 2023].

[4] Abdool Karim SS, Churchyard GJ, Karim QA, Lawn SD. HIV infection and tuberculosis in South Africa: An urgent need to escalate the public health response. Lancet 2009;374(9693):921–933; doi: 10.1016/s0140-6736(09)60916-8 19709731 PMC2803032

[5] Mollel EW, Todd J, Mahande MJ, Msuya SE. Effect of tuberculosis infection on mortality of HIV-infected patients in Northern Tanzania. Trop Med Health 2020;48:26; doi: 10.1186/s41182-020-00212-z 32355448 PMC7184680

[6] Goletti D, Weissman D, Jackson RW, et al. Effect of *Mycobacterium tuberculosis* on HIV replication. Role of immune activation. J Immunol 1996;157(3):1271–1278; doi: 10.4049/jimmunol.157.3.1271 8757635

[7] Toossi Z, Mayanja-Kizza H, Hirsch CS, et al. Impact of tuberculosis (TB) on HIV-1 activity in dually infected patients. Clin Exp Immunol 2001;123(2):233–238; doi: 10.1046/j.1365-2249.2001.01401.x 11207653 PMC1905977

[8] Bell LCK, Noursadeghi M. Pathogenesis of HIV-1 and *Mycobacterium tuberculosis* co-infection. Nat Rev Microbiol 2018;16(2):80–90; doi: 10.1038/nrmicro.2017.128 29109555

[9] Lawn SD, Pisell TL, Hirsch CS, et al. Anatomically compartmentalized human immunodeficiency virus replication in HLA-DR+ cells and CD14+ macrophages at the site of pleural tuberculosis coinfection. J Infect Dis 2001;184(9):1127–1133. 11598835 10.1086/323649

[10] Nakata K, Rom WN, Honda Y, et al. *Mycobacterium tuberculosis* enhances human immunodeficiency virus-1 replication in the lung. Am J Respir Crit Care Med 1997;155(3):996–1003; doi: 10.1164/ajrccm.155.3.9117038 9117038

[11] Kumawat K, Pathak SK, Spetz AL, et al. Exogenous Nef is an inhibitor of *Mycobacterium tuberculosis*-induced tumor necrosis factor-alpha production and macrophage apoptosis. J Biol Chem 2010;285(17):12629–12637; doi: 10.1074/jbc.M109.073320 20068037 PMC2857058

[12] Mwandumba HC, Russell DG, Nyirenda MH, et al. *Mycobacterium tuberculosis* resides in nonacidified vacuoles in endocytically competent alveolar macrophages from patients with tuberculosis and HIV infection. J Immunol 2004;172(7):4592–4598; doi: 10.4049/jimmunol.172.7.4592 15034077

[13] Patel NR, Zhu J, Tachado SD, et al. HIV impairs TNF-alpha mediated macrophage apoptotic response to *Mycobacterium tuberculosis*. J Immunol 2007;179(10):6973–6980. 17982088 10.4049/jimmunol.179.10.6973

[14] Sullivan ZA, Wong EB, Ndung'u T, et al. Latent and active tuberculosis infection increase immune activation in individuals co-infected with HIV. EBioMedicine 2015;2(4):334–340; doi: 10.1016/j.ebiom.2015.03.005 26114158 PMC4476549

[15] Toossi Z, Funderburg NT, Sirdeshmuk S, et al. Systemic immune activation and microbial translocation in dual HIV/tuberculosis-infected subjects. J Infect Dis 2013;207(12):1841–1849; doi: 10.1093/infdis/jit092 23479321 PMC3654743

[16] Kaufmann SH, Dorhoi A. Inflammation in tuberculosis: Interactions, imbalances and interventions. Curr Opin Immunol 2013;25(4):441–449; doi: 10.1016/j.coi.2013.05.005 23725875

[17] Roy Chowdhury R, Vallania F, Yang Q, et al. A multi-cohort study of the immune factors associated with *M. tuberculosis* infection outcomes. Nature 2018;560(7720):644–648; doi: 10.1038/s41586-018-0439-x 30135583 PMC6414221

[18] Carbone J, Gil J, Benito JM, et al. Increased levels of activated subsets of CD4 T cells add to the prognostic value of low CD4 T cell counts in a cohort of HIV-infected drug users. AIDS (London, England) 2000;14(18):2823–2829; doi: 10.1097/00002030-200012220-00003 11153663

[19] Deeks SG, Kitchen CM, Liu L, et al. Immune activation set point during early HIV infection predicts subsequent CD4+ T-cell changes independent of viral load. Blood 2004;104(4):942–947; doi: 10.1182/blood-2003-09-3333 15117761

[20] Giorgi JV, Liu Z, Hultin LE, et al. Elevated levels of CD38+ CD8+ T cells in HIV infection add to the prognostic value of low CD4+ T cell levels: results of 6 years of follow-up. The Los Angeles Center, Multicenter AIDS Cohort Study. J Acquir Immune Defic Syndr (1988) 1993;6(8):904–912. 7686224

[21] Hazenberg MD, Otto SA, van Benthem BH, et al. Persistent immune activation in HIV-1 infection is associated with progression to AIDS. AIDS 2003;17(13):1881–1888; doi: 10.1097/01.aids.0000076311.76477.6e 12960820

[22] Adekambi T, Ibegbu CC, Cagle S, et al. Biomarkers on patient T cells diagnose active tuberculosis and monitor treatment response. J Clin Invest 2015;125(5):1827–1838; doi: 10.1172/JCI77990 25822019 PMC4598074

[23] Day CL, Abrahams DA, Harris LD, et al. HIV-1 infection is associated with depletion and functional impairment of *Mycobacterium tuberculosis*-specific CD4 T cells in individuals with latent tuberculosis infection. J Immunol 2017;199(6):2069–2080; doi: 10.4049/jimmunol.1700558 28760884 PMC5624214

[24] Wilkinson KA, Oni T, Gideon HP, et al. Activation profile of *Mycobacterium tuberculosis*-specific CD4(+) T cells reflects disease activity irrespective of HIV status. Am J Respir Crit Care Med 2016;193(11):1307–1310; doi: 10.1164/rccm.201601-0116LE 27248590 PMC4910903

[25] Chetty S, Govender P, Zupkosky J, et al. Co-infection with *Mycobacterium tuberculosis* impairs HIV-Specific CD8+ and CD4+ T cell functionality. PLoS One 2015;10(3):e0118654; doi: 10.1371/journal.pone.0118654 25781898 PMC4363785

[26] Barham MS, Abrahams DA, Khayumbi J, et al. HIV infection is associated with downregulation of BTLA expression on *Mycobacterium tuberculosis*-specific CD4 T cells in active tuberculosis disease. Front Immunol 2019;10:1983; doi: 10.3389/fimmu.2019.01983 31497018 PMC6712065

[27] Parrish NM, Carroll KC. Role of the clinical mycobacteriology laboratory in diagnosis and management of tuberculosis in low-prevalence settings. J Clin Microbiol 2011;49(3):772–776; doi: 10.1128/jcm.02451-10 21177899 PMC3067741

[28] Finak G, McDavid A, Chattopadhyay P, et al. Mixture models for single-cell assays with applications to vaccine studies. Biostatistics 2014;15(1):87–101; doi: 10.1093/biostatistics/kxt024 23887981 PMC3862207

[29] Laidlaw BJ, Craft JE, Kaech SM. The multifaceted role of CD4(+) T cells in CD8(+) T cell memory. Nat Rev Immunol 2016;16(2):102–111; doi: 10.1038/nri.2015.10 26781939 PMC4860014

[30] Kornbluth RS. The emerging role of CD40 ligand in HIV infection. J Leukoc Biol 2000;68(3):373–382; doi: 10.1189/jlb.68.3.373 10985254

[31] Vanham G, Penne L, Devalck J, et al. Decreased CD40 ligand induction in CD4 T cells and dysregulated IL-12 production during HIV infection. Clin Exp Immunol 1999;117(2):335–342; doi: 10.1046/j.1365-2249.1999.00987.x 10444266 PMC1905331

[32] Collins DR, Gaiha GD, Walker BD. CD8(+) T cells in HIV control, cure and prevention. Nat Rev Immunol 2020;20(8):471–482; doi: 10.1038/s41577-020-0274-9 32051540 PMC7222980

[33] Makedonas G, Betts MR. Living in a house of cards: Re-evaluating CD8(+) T-cell immune correlates against HIV. Immunol Rev 2011;239(1):109–124; doi: 10.1111/j.1600-065X.2010.00968.x 21198668 PMC3025661

[34] Migueles SA, Laborico AC, Shupert WL, et al. HIV-specific CD8+ T cell proliferation is coupled to perforin expression and is maintained in nonprogressors. Nat Immunol 2002;3(11):1061–1068; doi: 10.1038/ni845 12368910

[35] Migueles SA, Osborne CM, Royce C, et al. Lytic granule loading of CD8+ T cells is required for HIV-infected cell elimination associated with immune control. Immunity 2008;29(6):1009–1021; doi: 10.1016/j.immuni.2008.10.010 19062316 PMC2622434

[36] Migueles SA, Connors M. Success and failure of the cellular immune response against HIV-1. Nat Immunol 2015;16(6):563–570; doi: 10.1038/ni.3161 25988888

[37] Morou A, Palmer BE, Kaufmann DE. Distinctive features of CD4+ T cell dysfunction in chronic viral infections. Curr Opin HIV AIDS 2014;9(5):446–451; doi: 10.1097/COH.0000000000000094 25023623 PMC4231289

[38] Adekambi T, Ibegbu CC, Cagle S, et al. High frequencies of Caspase-3 expressing *Mycobacterium tuberculosis*-specific CD4(+) T cells are associated with active tuberculosis. Front Immunol 2018;9:1481; doi: 10.3389/fimmu.2018.01481 29983703 PMC6026800

[39] Gaiha GD, McKim KJ, Woods M, et al. Dysfunctional HIV-specific CD8+ T cell proliferation is associated with increased caspase-8 activity and mediated by necroptosis. Immunity 2014;41(6):1001–1012; doi: 10.1016/j.immuni.2014.12.011 25526311 PMC4312487

[40] Oni T, Burke R, Tsekela R, et al. High prevalence of subclinical tuberculosis in HIV-1-infected persons without advanced immunodeficiency: Implications for TB screening. Thorax 2011;66(8):669–673; doi: 10.1136/thx.2011.160168 21632522 PMC3142344

