# Supplementary figures and images for: Active Tuberculosis Is Associated with Depletion of HIV-Specific CD4 and CD8 T Cells in People with HIV
Source: AIDS Res Hum Retroviruses. 2024 Jul 11;40(7):417–27. doi: 10.1089/aid.2023.0088 (PMC11295841; doi:10.1089/aid.2023.0088)

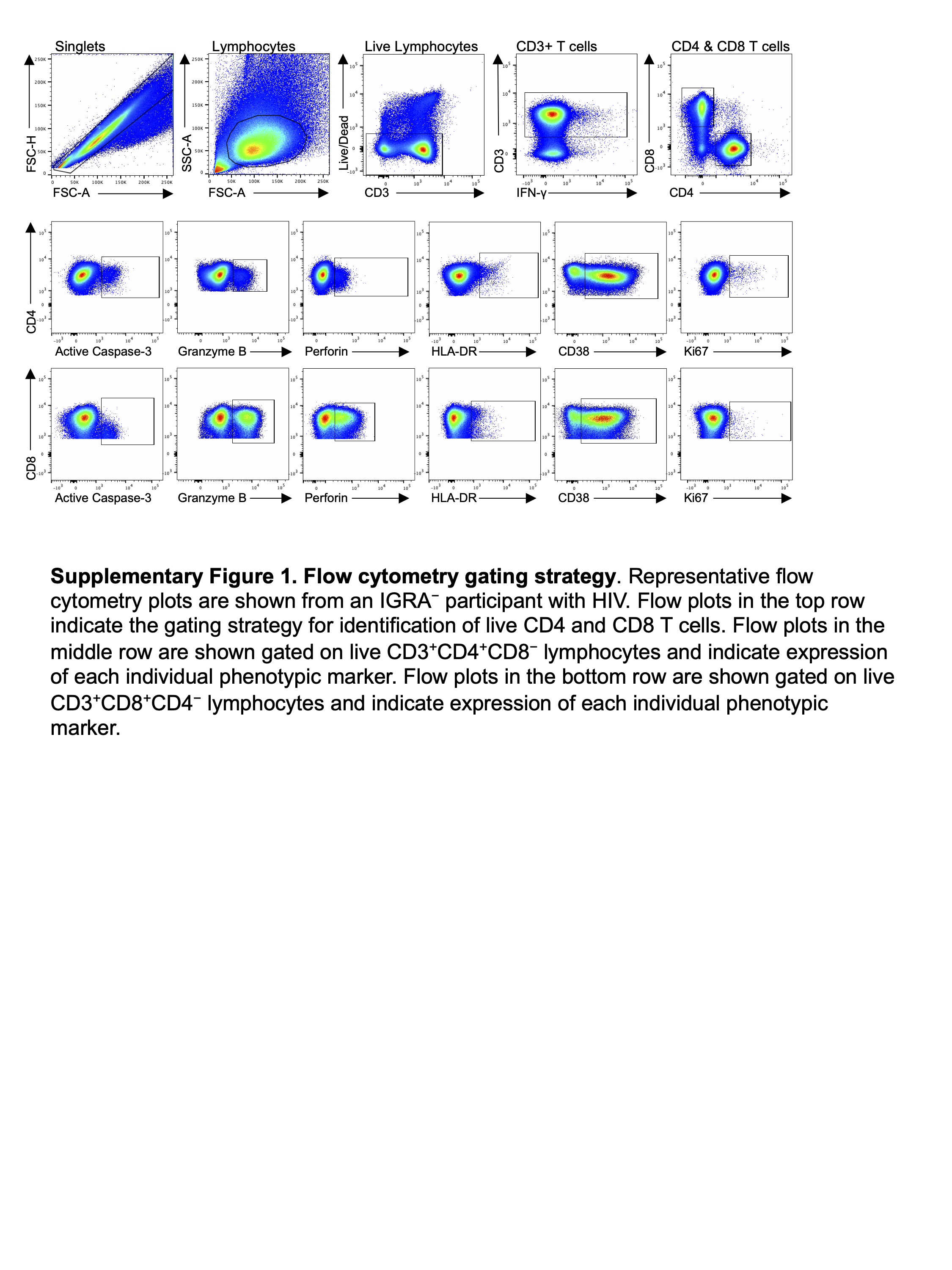

Supplement: Supplementary Figure S1 [file aid.2023.0088_suppl_figures1.tiff]
